# Optical molecular imaging can differentiate metastatic from benign lymph nodes in head and neck cancer

**DOI:** 10.1038/s41467-019-13076-7

**Published:** 2019-11-06

**Authors:** Naoki Nishio, Nynke S. van den Berg, Stan van Keulen, Brock A. Martin, Shayan Fakurnejad, Nutte Teraphongphom, Stefania U. Chirita, Nicholas J. Oberhelman, Guolan Lu, Crista E. Horton, Michael J. Kaplan, Vasu Divi, A. Dimitrios Colevas, Eben L. Rosenthal

**Affiliations:** 1 Department of Otolaryngology-Head and Neck Surgery, Stanford University School of Medicine, 900 Blake Wilbur Drive, Stanford, CA 94305 USA; 2 Department of Otorhinolaryngology, Nagoya University Graduate School of Medicine, 65 Tsurumai-cho, Showa-ku, Nagoya, 466-8550 Japan; 3 Department of Oral and Maxillofacial Surgery/Oral Pathology, VU University Medical Center/Academic Centre for Dentistry Amsterdam (ACTA), De Boelelaan 1117, 1081 HV Amsterdam, The Netherlands; 4 Department of Pathology, Stanford University School of Medicine, 300 Pasteur Drive, Stanford, CA 94305 USA; 5 Department of Medicine, Division of Medical Oncology, Stanford University School of Medicine, 875 Blake Wilbur Drive, Stanford, CA 94305 USA

**Keywords:** Cancer imaging, Head and neck cancer, Surgical oncology

## Abstract

Identification of lymph node (LN) metastasis is essential for staging of solid tumors, and as a result, surgeons focus on harvesting significant numbers of LNs during ablative procedures for pathological evaluation. Isolating those LNs most likely to harbor metastatic disease can allow for a more rigorous evaluation of fewer LNs. Here we evaluate the impact of a systemically injected, near-infrared fluorescently-labeled, tumor-targeting contrast agent, panitumumab-IRDye800CW, to facilitate the identification of metastatic LNs in the ex vivo setting for head and neck cancer patients. Molecular imaging demonstrates a significantly higher mean fluorescence signal in metastatic LNs compared to benign LNs in head and neck cancer patients undergoing an elective neck dissection. Molecular imaging to preselect at-risk LNs may thus allow a more rigorous examination of LNs and subsequently lead to improved prognostication than regular neck dissection.

## Abstract (web summary)

Imaging metastatic disease burden in lymph nodes is important for surgical decision making. Here, the authors use an antibody-dye conjugate to distinguish between metastatic and benign lymph nodes in head and neck cancer.

## Introduction

In patients with solid tumors the presence of lymph node (LN) metastases is considered an important negative prognostic factor for survival^1,2^. Although multiple imaging modalities are available for preoperative staging, including positron emission tomography combined with computed tomography (PET/CT) and magnetic resonance imaging (MRI), they are suboptimal in detecting occult LN (micro-) metastasis. In squamous cell carcinoma of the head and neck (HNSCC), there is up to a 30% chance of presence of occult nodal metastasis at the time of surgery, despite clinical and radiographic evidence to support the absence of tumor in the LNs of the neck (cN0)^2^. Consequently, patients with early stage disease and no clinical and radiographic evidence of regional LN metastasis routinely undergo an elective neck dissection^3–5^.

Histopathological evaluation of neck dissection specimens relies on the identification and harvesting of LNs from the received neck specimen^6^. Identification of metastatic LNs within the fibro-adipose tissue of the neck specimen remains a challenge, despite that gross and histologic examination of the neck specimen is critical for accurate staging and guides the extent of adjuvant therapy and prognosis^7^. The current workflow necessitates that at pathology as many LNs as possible are harvested from the neck specimen, which are then all macro- and microscopically inspected for the presence of metastasis. This work load has been further increased by recent quality standards endorsed by the American College of Surgery, which increased the standards for number of LNs required for pathological evaluation.

Advancement in new technologies in near-infrared (NIR) imaging hardware and molecular contrast agents have resulted in a significant number of new intraoperative imaging contrast agents entering clinic trials. The potential value in management of the primary tumor has been emphasized with improved sensitivity and specificity of (microscopic) tumor detection in the operative setting^8–11^, but the benefit of these technologies in detecting regional LN metastasis has undergone limited evaluation^12^. Although there are currently 13 open trials listed on ClinicalTrials.gov that evaluate the use of this technology, the potential impact on LN assessment at pathology has largely been ignored.

The current study evaluates the impact of a systemically injected, near-infrared fluorescently-labeled, tumor-targeting contrast agent, panitumumab-IRDye800CW, to facilitate the identification of metastatic LNs in the ex vivo setting for head and neck cancer patients. Molecular imaging demonstrates a significantly higher mean fluorescence signal in metastatic LNs compared to benign LNs in head and neck cancer patients undergoing an elective neck dissection. Molecular imaging to preselect at-risk LNs may thus allow a more rigorous examination of LNs and subsequently lead to improved prognostication than regular neck dissection.

## Results

### Summary of study design

A single center, non-randomized, phase I study was performed in 24 patients with biopsy-proven HNSCC, of which 22 patients received surgical resection of the primary tumor and a unilateral or bilateral neck dissection. Patients received an infusion with panitumumab-IRDye800CW 1–5 days prior to surgery at a range of doses: low (<0.5 mg kg^−1^), middle (≥0.5– < 0.75 mg kg^−1^) and high (≥0.75 mg kg^−1^). On the day of surgery intraoperative, NIR fluorescence imaging was performed to assess cervical LNs in vivo using an open-field device. After surgical resection, the neck dissection specimens were imaged in a closed-field fluorescence device and subsequently sent for pathology and processed as per standard of care. In collaboration with a board-certified pathologist (B.A.M), molecular imaging results were correlated to (histo-) pathological findings^13^.

**Fig. 1:**
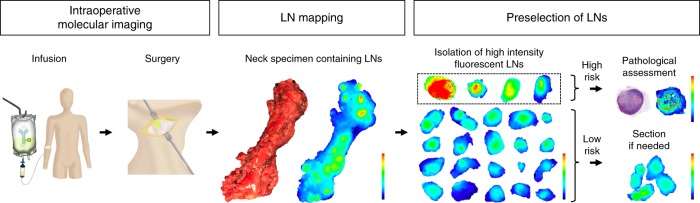
Workflow of Fluorescence Imaging-Based LN Preselection. Following panitumumab-IRDye800CW infusion 1–5 days prior to the day of surgery, on the day of surgery, the neck will be operated on after which the neck specimen is collected and imaged with a closed-field imaging device. With the fluorescence guide of the neck specimen, LN grossing is performed as per standard of care. Fluorescence-imaging-based preselection using the fluorescence signal is performed to select LNs at risk of metastasis. Upon showing the feasibility of our approach, we propose that only at-risk LNs, which are characterized by a high mean fluorescence intensity (MFI) and signal-to-background ratio (SBR), have to undergo thorough evaluation at pathology, meaning an hematoxylin and eosin staining, and a closed-field fluorescence image is obtained to correlate fluorescence-imaging-based findings to (histo-) pathology. Low-risk LNs would not require any further evaluation. *LN* lymph node

Based on the results of the current study, we propose an enhanced workflow using pathological molecular imaging to segregate high-risk (metastatic) from low-risk (benign) LNs, as demonstrated in Fig. 1.

### Patient characteristics

Table 1 shows the characteristics of the 22 patients who received surgical resection of the primary tumor and a unilateral or bilateral neck dissection. Preoperative MRI imaging was performed in 19 patients (86.4%), and an ^18^F-FDG-PET/CT scan was acquired in 16 patients (72.7%). A CT scan was performed in seven patients (31.8%). Clinical N stage (cN) was N0 in 14 patients (63.6%), N1 in five patients (22.7%), and N2 in three patients (13.6%).

**Table 1:** Patient demographics and pathological characteristics

Characteristics	N (%)	Average (range)
Number of patients	22 (100)	
Gender, male	16 (72.7)	
Age (y)		60 (32–85)
Panitumumab-IRDye800CW dose (mg kg^−1^)		0.67 (0.26–1.05)
Time of infusion-to-surgery (h)		57 (17–120)
Diagnostic imaging modality		
MRI	19 (86.4)	
^18^F-FDG-PET/CT	16 (72.7)	
CT	7 (31.8)	
Primary tumor site		
Oral cavity	20 (90.9)	
Hypopharynx	1 (4.5)	
Larynx	1 (4.5)	
Tumor size (mm)		34 (6–55)
Clinical N-stage		
N0	14 (63.6)	
N1	5 (22.7)	
N2	3 (13.6)	
Pathological N-stage		
N0	13 (59.1)	
N1	1 (4.5)	
N2	8 (36.3)	
Surgical procedure		
Tumor resection + unilateral neck dissection	14 (63.6)	
Tumor resection + bilateral neck dissection	8 (36.4)	

*MRI* magnetic resonance imaging, ^*18*^*F* fluoride 18, *FDG* fluorodeoxyglucose, *PET/CT* positron emission tomography combined with computed tomography, *CT* computed tomography

The average panitumumab-IRDye800CW dose given was 0.67 mg kg^−1^ (range 0.26–1.05 mg kg^−1^) and the average time of infusion-to-surgery was 2 days (range 17–120 h). Patients were followed for 30 days post-study drug infusion and adverse event data was collected on day 0, day of surgery, day 15, and day 30. Adverse events were classified according to the National Cancer Institute Common Terminology Criteria v4.0. No adverse events were reported that were found to be related to the study. Furthermore, no abnormalities were found in general physical exam, Karnofsky performance status, metabolic panels, complete blood count, serum chemistry, prothrombin/partial thromboplastin times, thyroid stimulating hormone levels, and ECGs.

At surgery, a total of 30 neck dissection specimens were obtained; 14 patients (63.6%) underwent a unilateral neck dissection and a bilateral neck dissection was performed in eight patients (36.4%). Following (histo-) pathology, a total of 1012 LNs (39 metastatic LNs and 973 benign LNs) were identified, averaging 37.5 LNs per neck (range 12–72 LNs), which is consistent with our institutional average of 36 LNs per neck (internal quality data, unpublished). Of the total number of LNs collected, 946 LNs (93.5%) were classified as small LNs, with a maximal LN diameter <10 mm.

### Discriminating metastatic from benign LNs

**Fig. 2:**
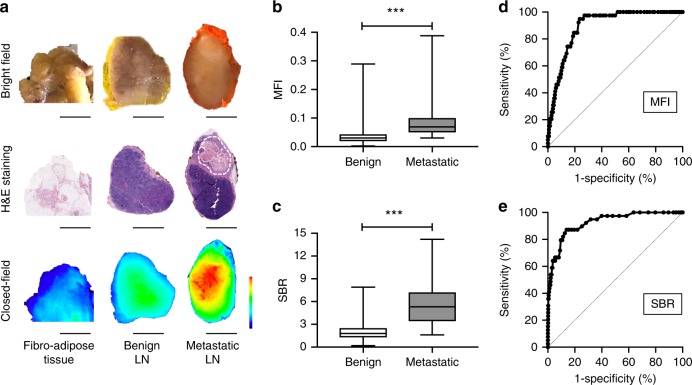
Panitumumab-IRDye800CW Distinguishes Metastatic and Benign LNs. **a** Fluorescence-imaging data was calculated as mean fluorescence intensity (MFI) and signal-to-background ratio (SBR) and then compared with histopathology. Dotted line: tumor region. Scale bars = 5 mm. **b** Quantitative fluorescence signal analysis showed a significant higher MFI in metastatic LNs vs. benign LNs (*p* < 0.001). **c** Using fibro-adipose tissue as background signal, the SBR was >2-fold higher in metastatic LNs compared to the SBR in benign LNs (*p* < 0.001). **d**, **e** Receiver operating characteristic curve (ROC) curve evaluation showed that high areas under the curve (AUCs) were obtained for MFI (AUC = 0.89, 95% confidence interval (CI) 0.86–0.92) and SBR (AUC = 0.93, 95% CI; 0.89–0.97). Data are presented as boxplots, where the whiskers indicate the minimum and maximum value, and lower and upper hinges correspond to the upper and lower quartiles, center line to the median. Figure 2 (b),(c): Benign (*n* = 973) and metastatic (*n* = 39). *P*-values were determined via a two-tailed Mann–Whitney U test. (*p < 0.05; **p < 0.01; ***p < 0.001; NS not significant) Source data are provided as a Source Data file. *LN* lymph node

To evaluate the sensitivity and specificity of pathological molecular imaging using an anti-EGFR fluorescent contrast agent for the identification of metastatic LNs, we performed closed-field fluorescence imaging of the neck specimens and thereafter of the individually dissected LNs prior to processing for (histo-) pathological analysis. Fluorescence-imaging data were calculated as mean fluorescence intensity (MFI) and signal-to-background ratio (SBR) and then compared to histopathology (Fig. 2a). Fluorescence signal intensity analysis showed a significantly higher MFI in metastatic LNs vs. benign LNs, 0.099 ± 0.014 vs. 0.036 ± 0.001, respectively (mean ± standard deviation (SD); Mann–Whitney U-test, *p* < 0.001; Fig. 2b). Using fibro-adipose tissue for reference background signal, the mean SBR was >2-fold higher in metastatic LNs compared to the mean SBR in benign LNs, 5.8 ± 0.5 vs. 2.0 ± 0.1 (Mann–Whitney U-test, *p* < 0.001; Fig. 2c).

The optimal threshold value for MFI was found to be 0.044 at which a 94.9% sensitivity and a 76.4% specificity was reached [likelihood ratio (LR) 4.0]. Here, the negative predictive value (NPV) of this technique was 99.7% and the positive predictive value (PPV) was 13.9%.

The optimal threshold value for SBR was 3.0, whereby an 87.2% sensitivity and 86.1% specificity (LR 6.2) was reached, with an NPV and PPV of 99.4% and 20.1%, respectively. Receiver operating characteristic (ROC) curve evaluation showed the area under the curve (AUC) obtained for MFI was 0.89 (95% confidence interval (CI) 0.86–0.92) and the AUC for SBR was 0.93 (95% CI 0.89–0.97), suggesting potential clinical value (Fig. 2d, e).

Because the PPV remained relatively low for the single evaluation techniques, we evaluated a combined threshold of MFI ≥ 0.044 and SBR ≥ 3.0, which resulted in a PPV of 36.2% consistent with a sensitivity of 84.6% and specificity of 94.0% (Table 2). Importantly, this combined MFI and SBR threshold method allowed us to preselect 91 LNs (9.0%) from the total of 1012 collected LNs, compared to 267 LNs (26.4%) in MFI threshold method and 169 LNs (16.7%) in SBR threshold method. Finally, using the combined MFI and SBR threshold method, 915/1012 LNs were scored true negative (fluorescence negative, histopathologically confirmed benign LN), 33 LNs as true positive (fluorescence positive, histopathologically confirmed metastatic LN), 58 LNs as false positive (fluorescence positive, histopathologically confirmed benign LN), and 6 LNs as false negative (fluorescence negative, histopathologically confirmed metastatic LN).

**Table 2:** Diagnostic statistics for determining the threshold for LN preselection

Threshold values	Sensitivity	Specificity	Positive predictive value	Negative predictive value	Accuracy	Preselected number/Total number of LNs (%)
MFI ≥ 0.044	94.9%	76.4%	13.9%	99.7%	77.1%	267/1012 (26.4)
SBR ≥ 3.0	87.2%	86.1%	20.1%	99.4%	86.2%	169/1012 (16.7)
MFI ≥ 0.044 + SBR ≥ 3.0	84.6%	94.0%	36.2%	99.3%	93.7%	91/1012 (9.0)

*MFI* mean fluorescence intensity, *SBR* signal-to-background ratio, *LN* lymph node

To determine the clinical impact of the six false negative LNs (0.6%), we evaluated the staging in these four patients. All four patients presented with multiple metastatic LNs, none larger than 6 cm and none with extranodal extension (staged as pN2); fluorescence imaging detected ≥2 LNs in each patient, and therefore the presence of additional small metastatic LNs did not impact staging.

### Identification of metastases in small LNs

**Fig. 3:**
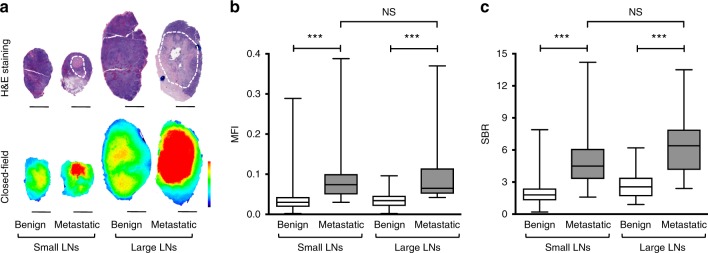
Fluorescence Signal Intensities of Small and Large Metastatic LNs are Similar. **a** Representative hematoxylin and eosin (H&E) staining and fluorescence image of small (<10 mm) and large (≥10 mm) LNs. Dotted line: tumor region. Scale bars = 5 mm. **b**, **c** Mean fluorescence intensity (MFI) and signal-to-background ratio (SBR) of metastatic and benign LNs are significantly different when assessing small or large LNs only. However, no difference in MFI and SBR are found for metastatic small and large LNs. Data are presented as boxplots, where the whiskers indicate the minimum and maximum value, and lower and upper hinges correspond to the upper and lower quartiles, center line to the median. Figure 3(b), (c): In small LNs (*n* = 946), benign (*n* = 924), and metastatic (*n* = 22). In large LNs (*n* = 66), benign (*n* = 49), and metastatic (*n* = 17). *P*-values were determined via a two-tailed Mann–Whitney U test. (*p < 0.05; **p < 0.01; ***p < 0.001; NS, not significant) Source data are provided as a Source Data file. *LN* lymph node

In contrast to small (<10 mm) LNs, large LNs are readily identified intraoperatively and at pathology while grossing the neck specimen. To assess whether our method could identify small metastatic LNs, we evaluated MFI and SBR separately for these small LNs (*n* = 946, Fig. 3a). The fluorescence signal intensity of metastatic LNs was >2-fold higher than that of benign LNs (Fig. 3b, c) suggesting that pathologic molecular imaging can detect metastatic disease in small LNs. An 81.8% sensitivity and a 94.0% specificity was reached with a NPV of 99.5% for small LNs (Table 3).

**Table 3:** Cumulative statistics for the total number of harvested LNs

	Total LNs	Small LNs (<10 mm)	Large LNs (≥10 mm)	*p*-value
Number of LNs	1012	946	66	
Metastatic				
True positive	33	18	15	NS
False negative	6	4	2	
Benign				
False positive	58	55	3	NS
True negative	915	869	46	
Sensitivity	84.6%	81.8%	88.2%	
Specificity	94.0%	94.0%	93.9%	
Positive predictive value	36.2%	24.7%	83.3%	
Negative predictive value	99.3%	99.5%	95.8%	
Accuracy	93.7%	93.8%	92.4%	

*NS* not significant, *LN* lymph node

### Discriminating metastatic from large reactive LNs

Large LNs may be reactive (benign) or metastatic, and often complicate surgical decision making. We evaluated the fluorescence signal intensity between metastatic and benign LNs that were greater than 10 mm. For large LNs (*n* = 66), the fluorescence signal intensity from metastatic LNs was almost three times that of benign LNs (Fig. 3b, c), suggesting that our proposed approach could distinguish metastatic LNs from the other (reactive) LNs greater than 10 mm. A sensitivity of 88.2% and a specificity of 93.9% was reached with an NPV of 95.8% (Table 3). A much higher PPV was obtained in large LNs (83.3%) compared to small LNs (24.7%), likely reflecting the increased prevalence of disease (higher pre-test probability) among larger LNs.

### Higher dose positively correlates with false positive rate

**Fig. 4:**
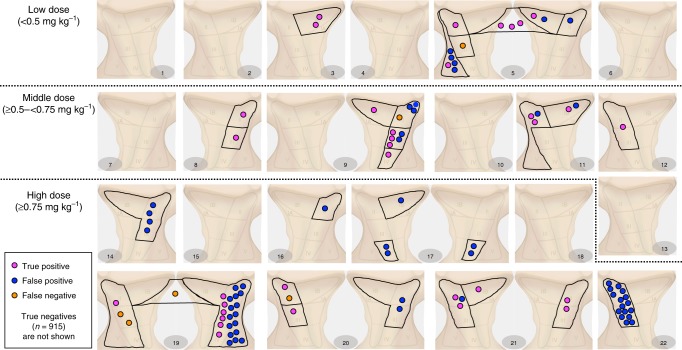
Patient-based Distribution of True Positive, False Positive and False Negative LNs. A total of 1012 LNs was collected from 22 patients of which 915 were true negatives (not shown). The number of true positive, false positive and false negative LNs is shown on a per patient base. Definitions: True positive = fluorescence positive, histopathologically confirmed metastatic LN; True negative = fluorescence negative, histopathologically benign LN; False positive = fluorescence positive, histopathologically benign LN; False negative = fluorescence negative, histopathologically confirmed metastatic LN. *LN* lymph node

**Fig. 5:**
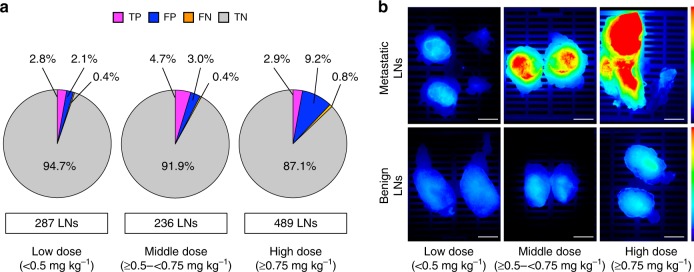
Effect of the Antibody-Dye Dose on the Find Rate of False Positive LNs. **a** Rates of true positive, false positive, false negative and true negative LNs over three groups by weight adjusted dose. *TP* true positive (fluorescence positive, histopathologically confirmed metastatic LN); *TN* true negative (fluorescence negative, histopathologically benign LN); *FP* false positive (fluorescence positive, histopathologically benign LN); *FN* false negative (fluorescence negative, histopathologically confirmed metastatic LN). Source data are provided as a Source Data file. **b** Representative fluorescence images of metastatic and benign LNs for the three dose groups. Scale bars = 5 mm. *LN* lymph node

To study the impact of panitumumab-IRDye800CW dose on the false positive and false negative rate, we divided the 22 studied patients over three dose groups: < 0.5 mg kg^−1^ (low dose, *n* = 6), ≥0.5- < 0.75 mg kg^−1^ (middle dose, *n* = 7), and ≥0.75 mg kg^−1^ (high dose, *n* = 9). The distribution of true positive, false positive and false negative LNs across the 22 patients is shown in Fig. 4 by the combined MFI and SBR threshold method described above. Not surprisingly the fluorescence signal intensity of metastatic LNs in the low dose group was significantly lower compared to the middle or high dose group (Kruskal-Wallis test with a Dunn’s multiple comparison test, *p* < 0.01). The SBR was not found to vary with dose. Of the total of 58 false positive LNs, 45 LNs (77.6%) were found in the high dose group. This resulted in a false positive rate of 9.2% in the high dose group vs. 2.1% in the low dose group and 3.0% in the middle dose group (Fig. 5a). Furthermore, PPVs in the low and middle dose group were much higher compared to the PPV in the high dose group (57.1%, 61.1% vs. 23.7%; Supplementary Table 1). Optimal LN preselection results were obtained in the middle dose group (≥0.5– < 0.75 mg kg^−1^) where a sensitivity of 91.7% and a specificity of 96.9% with an NPV of 99.5% was found. Representative case examples are shown in Fig. 5b.

### Fluorescence signal intensity to segregate high-risk LNs

**Fig. 6:**
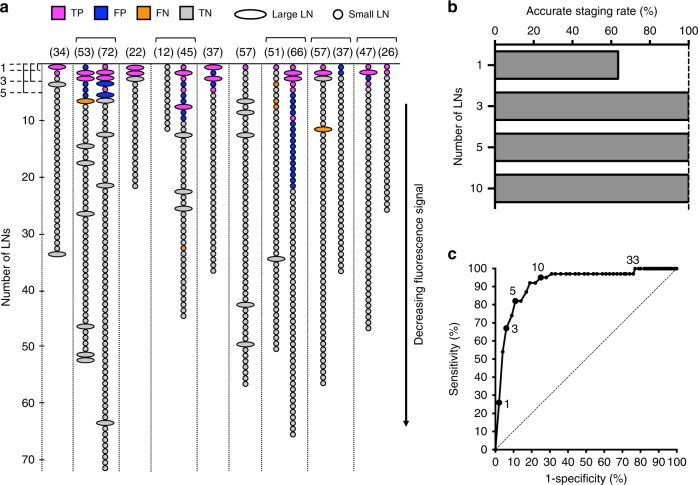
Fluorescence Signal Intensity-based Ranking of Dissected LNs per Neck. **a** Fluorescence signal intensity-based ranking of small and large LNs in the nine patients with pathologically positive necks. *TP* true positive (fluorescence positive, histopathologically confirmed metastatic LN); *TN* true negative (fluorescence negative, histopathologically benign LN); *FP* false positive (fluorescence positive, histopathologically benign LN); *FN* false negative (fluorescence negative, histopathologically confirmed metastatic LN). **b** Using the first three LNs with the highest fluorescence signal would provide accurate staging in all patients. **c** Sensitivity and specificity based on the number of LNs per neck that need to be assessed by pathology. Source data are provided as a Source Data file. *LN* lymph node

To identify the minimum number of LNs that would be required for histopathological assessment to achieve accurate staging, we ranked the LNs by absolute fluorescence signal (as defined by MFI), per neck. We mapped all of the LNs harvested from the 14 neck dissections in the nine patients with pathologically positive LNs (five patients underwent a bilateral neck dissection, Fig. 6a). Segregation of the LNs by decreasing MFI allowed for accurate staging of the neck by histological sectioning of only three LNs with the highest fluorescent signal (Fig. 6b), with high specificity (94.0%). For each patient, this method identified sufficient metastatic LNs to establish the presence of larger metastases (>3 cm, >6 cm) and determine focality (single vs. multiple LNs), laterality, and extranodal extension status, providing all the data necessary for accurate American Joint Committee on Cancer (AJCC) staging. While on an individual LN basis the sensitivity of 66.7% was relatively low when evaluating only three LNs per neck (Fig. 6c), the data suggests that the sensitivity of detecting metastasis could be significantly increased by analysis of additional LNs, but with a resulting decrease in specificity (i.e., more false positive LNs identified). Although evaluation of only three LNs successfully staged 100% of the patients, harvesting and evaluation of up to 10 LNs per neck led to a further improved sensitivity of 94.8%, though at the cost of losing specificity (decreased to 75.1%).

### Assessment of false positive LNs

To confirm that false positive LNs were truly benign and not related to sampling errors, additional sections were obtained from 28 false positive LNs from six neck specimens from five patients that were staged pathological N0 (pN0). From each formalin-fixed paraffin-embedded LN, three additional sections were collected at 150 µm intervals. Additional sectioning revealed that one out of 28 (3.6%) of the false positive LNs was positive for tumor (micro-metastasis). As a control, additional sections were obtained from patient-matched true negative LNs (*n* = 28), of which all were found benign on re-evaluation.

### Antibody-dye distribution in metastatic and benign LNs

**Fig. 7:**
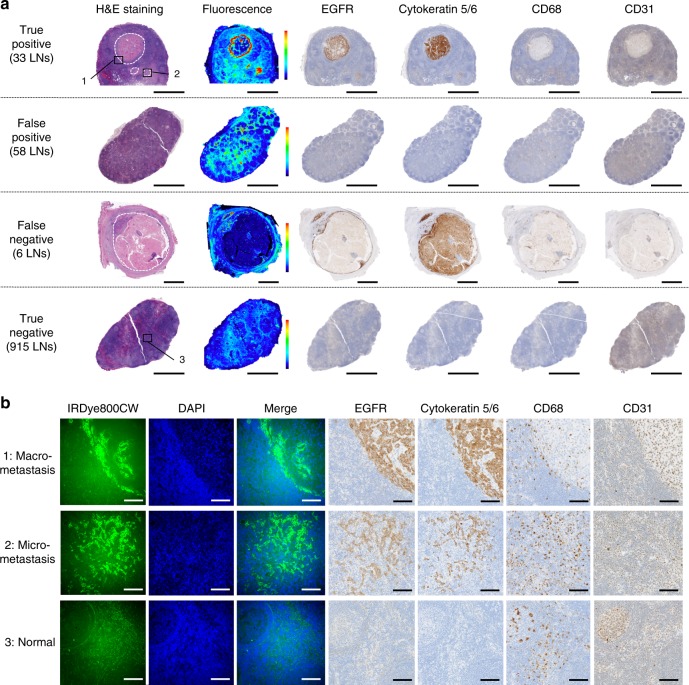
Panitumumab-IRDye800CW Distribution and Correlation to Immunohistochemistry. **a** Hematoxylin and eosin (H&E) staining, near-infrared fluorescence image and EGFR, cytokeratin 5/6, CD68 and CD31 immunohistochemistry results of a 4 µm cut section of representative true positive, false positive, false negative and true negative LNs. Dotted line: tumor region. Scale bars = 5 mm. **b** Representative microscopic fluorescence images of panitumumab-IRDye800CW (IRDye800CW) and EGFR immunohistochemistry in macro-metastasis (upper row), micro-metastasis (middle row) and uninvolved LN tissue (bottom row). Scale bars = 100 µm. *EGFR* epidermal growth factor receptor, *DAPI* 4′,6-diamidino-2-phenylindole (cell nucleus staining), *LN* lymph node

To assess the distribution of the fluorescent contrast agent on the microscopic level, we compared the fluorescence-imaging results to expression of EGFR, cytokeratin 5/6, CD68, and CD31 by performing immunohistochemistry of metastatic LNs (true positive, false negative) and benign LNs (false positive and true negative). In benign LNs, fluorescence was detected throughout the subcapsular and trabecular sinuses but not in the follicular regions and fluorescence did not correspond with the very low levels of EGFR expression (Fig. 7a). The number of false positive LNs dramatically increased with dose increases (Fig. 5 and Supplementary Table 1), suggesting that the fluorescent antibody migrated through LNs by vascular flow and/or non-specific lymphatic drainage. In metastatic LNs, the fluorescence signal was found in the peripheral aspects of macro-metastases and was well distributed within the micro-metastatic foci of tumor. Interestingly, in the false negative LNs, it appeared that the tumor was large enough to occlude lymphatic flow to the LNs, which was associated with a reduced fluorescence signal. Microscopic analysis of metastatic LNs demonstrated a clear correlation between EGFR expression and panitumumab-IRDye800CW uptake on the tumor cell membranes in macro-metastases and micro-metastases (Fig. 7b). Taken together, this data suggests that panitumumab-IRDye800CW migrates non-specifically through regional LNs and is capable of binding to tumor when present.

## Discussion

Surgical imaging for the detection of the primary tumor has rapidly evolved in the United States with many optical contrast agents being evaluated in clinical trials to improve negative margin rates during surgical resection^8,13,14^. As techniques in pathological molecular imaging improve for primary tumor evaluation^15,16^, there are distinct advantages that can be translated to assessment of regional disease and improvement of the pathology workflow as well.

It is well known that embedding high numbers of LNs results in the overload of labor, space, and use of equipment and supplies, all of which increase healthcare costs^17^. Moreover, methods for processing of specimens in surgical pathology have not changed in the past half century. We demonstrate that pathological molecular imaging following a systemic injection of an anti-EGFR imaging agent can guide ex vivo assessment of the resected nodal tissue based on fluorescence signal intensity (MFI) and signal-to-background ratio (SBR). We found that pathologically metastatic LNs could be successfully segregated from benign LNs by means of fluorescence assessment; when LNs were preselected based on MFI and SBR, metastatic LNs could be identified with high sensitivity (84.6%), specificity (94.0%), and NPV (99.3%) and acceptable PPV (36.2%). If prospective studies confirm this approach, our proposed LN assessment workflow (Fig. 1) could result in a 90% reduction of the number of LNs that have to undergo pathological processing and histological examination.

Although a combination of direct visualization and palpation is considered the gold standard by pathologists for detecting and harvesting LNs from neck dissection specimens, this technique is time consuming, can miss smaller LNs, and is subject to high interobserver variability, limiting standardization within institutions and across medical centers^18^. To improve nodal yield and to increase detection of small LNs, fat clearance can be performed^19^, but this technique requires a chemical agent to dissolve the fat tissue around the LNs, and thus is time consuming, expensive and potentially hazardous. Local or intravenous/intra-arterial injections of methylene blue and indocyanine green have been used to improve LN identification at pathology, but due to poor tumor specificity this has never become routinely implemented; sensitivities and specificities achieved with this approach vary between 52–94% and 76–78%, respectively^20–22^. Most importantly, none of the current techniques discriminates between metastatic LNs and benign LNs. Our proposed method, whereby anti-EGFR-based fluorescence molecular imaging allows for localization and segregating of LNs that are at the highest risk of harboring metastasis, provides an opportunity to ease the process of LN assessment at pathology. In the same way that sentinel LN mapping selects LNs most likely to harbor metastatic disease, pathological molecular imaging selects high-risk LNs rather than requiring complete processing of all the LNs. However, in contrast to sentinel LN mapping which requires a re-operation when metastatic LNs are found, molecular imaging allowed 95% sensitivity for detection of all metastatic LNs with a total of 10 LNs per neck required for histologic processing. To confirm that false-positive LNs were not a result of sampling error, 28 false positive (and patient-matched true negative) LNs were sectioned an additional 0.5 mm. The additional re-cutting revealed that 3.6% of the false positive LNs were found to have micro-metastasis, but none of the true negative LNs. Importantly, this new finding would have changed nodal staging in one patient.

The test performance characteristics of this molecular imaging technique are inherently related to the distribution of therapeutic antibodies in LNs after systemic administration, which is poorly understood in humans. In our study, microscopic imaging of panitumumab-IRDye800CW strongly corresponded with metastatic deposits within LNs that showed EGFR expression, and accumulation of panitumumab-IRDye800CW was predominantly found at the periphery of the metastasis, despite EGFR expression throughout the metastatic deposit (Fig. 7). This suggests that panitumumab-IRDye800CW binds to tumor nests and concentrates at the periphery during passive lymphatic flow, a process that would be less effective when the afferent lymphatic vessels are occluded by an expanding tumor mass (Fig. 7—false negative). In benign LNs on the other hand, panitumumab-IRDye800CW signal is most likely the result of non-specific accumulation, as demonstrated by the dose-dependent increase in fluorescent antibody-complex detection in the lymphatic sinuses despite the absence of EGFR expression in those LNs. This hypothesis is further supported by studies of anti-human epidermal growth factor receptor-2 (HER2) receptor monoclonal antibody (trastuzumab) which can extravasate and circulate through the lymphatic system after intravenous injection^23,24^.

Our current results may be significantly improved using the appropriate dose. Of the 58 false positive LNs, which were fluorescently positive but negative for tumor, 45 (77.6%) were found in the high dose cohort (>0.75 mg kg^−1^). Moreover, the PPV in this high dose group (23.7%) was much lower than in the low (57.1%) and middle dose groups (61.1%). This suggests that higher doses can increase non-specific drainage from the primary tumor to the tumor-draining LNs. Because optimal dosing for the primary tumor resection was previously established by us at 50 mg (0.5–0.75 mg kg^−1^) for head and neck cancer^13,25^, this will permit molecular imaging of the cervical LNs as well. It is important to recognize that optical imaging is highly dependent on the device hardware and software^26^. Although relative SBRs are more likely to be similar among the various devices that are being evaluated in clinical trials, results are expected to vary based on the intraoperative and pathologic imaging system used.

The process of LN preselection might be further improved by combining a radiotracer with fluorescence imaging to allow for a dual-modality in vivo and ex vivo molecular imaging strategy whereby preoperative, intraoperative and pathological processing can be all combined. A radiolabel would add the advantage of preoperative imaging and surgical guidance to deeper lying neck LNs, as compared to less than a centimeter of depth for optical agents^27,28^. Alternatively, a paired-agent molecular imaging method, whereby an untargeted imaging agent is administered alongside the targeted antibody, may be promising for assessing tumor burden in LNs based on preclinical data^29–31^. In a mouse model, this method demonstrated that the uptake of the untargeted agent could estimate non-specific uptake in LNs and combined with targeted agent the ratio of targeted vs. untargeted agent provided unprecedented sensitivity to cancer cell presence^30,32^. This paired-agent molecular imaging strategy is highly promising for metastatic LN identification; however, there are significant regulatory and financial barriers with implementation of a clinical trial with this strategy.

We describe the use of anti-EGFR molecular imaging for ex vivo nodal evaluation, which could improve standardization, efficiency and accuracy of histopathologic processing. Although not sufficient to justify the added cost of the imaging contrast agent and infusion, it would complement intraoperative imaging of the primary tumor, which we and others have shown to have enormous potential to improve quality of oncologic resections. Improvements of NIR open camera systems will likely also lead to the ability to accurately identify metastatic LNs in vivo^26^. In addition, optoacoustic imaging could improve depth of imaging by several centimeters^33–37^. The principles of optoacoustic imaging are similar to fluorescence imaging, but optoacoustics detects acoustic waves generated by the optical excitation of the fluorophore. Combining fluorescence imaging with optoacoustic imaging may thus allow for non-invasive detection of metastatic LNs in the in vivo setting to provide additional guidance for intraoperative identification of metastatic LNs.

Fluorescence molecular imaging using an anti-EGFR contrast agent can identify LNs at risk of harboring metastatic disease. Using fluorescence signal intensity to rank LNs in order of descending value, only 10% of harvested LNs required histopathological assessment to provide accurate regional staging. This strategy would decrease resource utilization, standardize pathological processing of LNs and improve diagnostic accuracy. Molecular imaging for pathological purposes should be carefully evaluated as the development of intraoperative molecular imaging for primary tumor resections becomes standard of care.

## Methods

### Clinical trial design

We performed a single center, non-randomized, prospective dose ranging study in patients with biopsy-proven HNSCC that were scheduled for surgical resection of curative intent. The primary objective of the study is to determine the safety profile of panitumumab-IRDye800CW. The secondary objective is to determine the efficiency of panitumumab-IRDye800CW to identify cancer compared to surrounding normal tissue.

The study protocol was approved by the Stanford University Administrative Panel on Human Subjects Research and the FDA (NCT02415881). The study was performed in accordance with the Helsinki Declaration of 1975 and its amendments, FDA’s ICH-GCP guidelines, and the laws and regulations of the United States. Written informed consent was obtained from all patients.

Adult patients with biopsy-proven, primary or recurrent head and neck squamous cell carcinoma scheduled to undergo standard-of-care surgery with curative intent were eligible. TNM stage was classified using the American Joint Committee on Cancer (AJCC) seventh edition criteria^38^. Exclusion criteria included a life expectancy of less than 12 weeks, a Karnofsky performance status <70%, prior infusion reactions to monoclonal antibodies, QT prolongation on screening electrocardiogram (ECG; >440 ms in males, and >450 ms in females), significant cardiopulmonary or liver disease, abnormal electrolyte values, and/or utilization of class IA or class III antiarrhythmic agents^13^.

Between 9/2016 and 3/2018, 24 patients were enrolled in this study. Patients were enrolled into four dose cohorts: 0.5 and 1.0 mg kg^−1^ in the weight-based dosing group and 25 and 50 mg in the fixed dosing group. Two patients were excluded from final analysis as they did not undergo a neck dissection.

### Panitumumab-IRDye800CW conjugation

Conjugation of panitumumab-IRDye800CW was performed under cGMP conditions, through NCI’s NeXT program^13^. Briefly, panitumumab (Vectibix; Amgen, Thousand Oaks, California, USA; 147 kDa) was conjugated to IRDye800CW-NHS via a 2-h incubation at 20 °C in the dark with a dye to protein ratio of 2.3:1. Quality control of the conjugate included analysis of drug product in sterile vial for particulates, and integrity of the sterilizing filter. Sterile vials were transported to Stanford University under temperature-controlled conditions and stored at the Stanford University Medical Center Investigational Pharmacy.

### Optical imaging of nodal specimens

Intraoperative imaging was performed pre-, inter-, and post-neck dissection using the Spy-Phi camera and IR9000 optical imaging platform modified for IRDye800CW fluorescence imaging (Novadaq, Burnaby, Canada). Immediately after resection, neck dissection specimens were re-imaged on the back table in the operation room using the same open-field imaging platform. Hereafter the neck specimens were transferred to a closed-field NIR closed-field optical imaging system (Pearl Triology; LI-COR Biosciences Inc., Lincoln, Nebraska, USA) and imaged. The closed-field imaging device was used for ex vivo neck specimen imaging because it has a wide dynamic range and ensures controlled imaging environment, including elimination of ambient light and fixed camera-tissue distance^39^. Subsequently the neck specimens were transported to pathology for standard of care processing. The neck specimens were formalin-fixed overnight after which they were re-imaged in the closed-field imaging device prior to grossing of the LNs. With the fluorescence guide of the neck specimen, LN grossing was performed as per standard of care. Grossed LNs greater than 5 mm diameter were bisected prior to embedding, per standard of care. Remainder fibro-adipose tissue from the neck specimens was also collected in additional cassettes. All cassetted LN samples, including the fibro-adipose tissues, were then re-imaged on the closed-field imaging device.

### Pathological assessment

After processing and paraffin embedding, 4 µm thick sections were sliced and stained with hematoxylin and eosin (H&E). The H&E slides were studied microscopically by a board-certificated pathologist, who was blinded to the fluorescence status, to confirm the number of LNs identified and the presence or absence of tumor cells in the LNs. The Odyssey imaging platform (LI-COR Biosciences Inc.) was used to determine fluorescence in slide-mounted sections obtained from formalin-fixed paraffin-embedded blocks.

To confirm the presence of EGFR, cytokeratin 5/6, CD68, and CD31 routine immunohistochemistry was performed with an autostainer (DAKO Link48 and PT link, Agilent Technologies Inc., Santa Clara, California, USA) using an anti-EGFR antibody (clone EP38Y, Thermo Fisher Scientific, Waltham, Massachusetts, USA), anti-cytokeratin 5/6 antibody (clone D5/16B4, Thermo Fisher Scientific), anti-CD68 antibody (clone KP1, Thermo Fisher Scientific), and anti-CD31 antibody (ab124432, Abcam, Cambridge, MA, USA). Stained slides were digitized using a whole slide scanner (Hamamatsu NanoZoomer 2.0-RS, Hamamatsu, Japan).

### Fluorescence microscopy

For fluorescence microscopy, selected tissue slides were deparaffinized, and the nuclei were counterstained with 4’,6-diamidino-2-phenylindole (DAPI, Prolong Diamond, Thermo Fisher Scientific). Stained slides were dried in the dark at 4 °C overnight. The slides were imaged using a custom set-up inverted digital fluorescence microscope (DM6B, Leica Biosystems, Wetzlar, Germany) equipped with a highly sensitive Leica DFC9000GT camera (4.2 M Pixel sCMOS camera), a metal halide LED light source (X-Cite 200DC, Excelitas Technologies, Waltham, Massachusetts, USA) for DAPI imaging, and a xenon arc lamp LB-LS/30 (Sutter Instrument, Novato, California, USA) for NIR imaging of IRDye800CW. Image acquisition and processing was done through LAS X software (Leica Biosystems).

### Statistical analysis

For quantitative analysis of the fluorescence-imaging-based data collected with the closed-field imaging device, mean fluorescence intensities (MFIs), defined as total counts/region of interest (ROI) pixel area, was calculated using a custom ROI generated for each LN using integrated instrument software (Image Studio; LI-COR Biosciences). Signal-to-background ratios (SBRs) were subsequently calculated by dividing LN MFI by the fibro-adipose tissue MFI. Descriptive statistics and figures were obtained using GraphPad Prism (Version 6.0c, GraphPad Software, La Jolla, California, USA). Data are presented as boxplots, where the whiskers indicate the minimum and maximum value, and lower and upper hinges correspond to the upper and lower quartiles, center line to the median. Sensitivity and specificity values of panitumumab-IRDye800CW for metastatic LN identification were calculated using ROC curves. Threshold values were determined based on Youden’s index using JMP (Version 10, SAS, Cary, North Carolina, USA). The likelihood ratio (LR) was defined by the following equation: LR = sensitivity/(1—specificity). Fluorescence intensities between the three different dose groups (<0.5 mg kg^−1^, ≥0.5- < 0.75 mg kg^−1^, and ≥0.75 mg kg^−1^) were compared using a Kruskal-Wallis test with a Dunn’s multiple comparison test as post hoc analyses. Fluorescence intensities between pathologically metastatic and benign LNs within each dose group was analyzed with a Mann–Whitney U-test. All data are presented as mean or mean ± standard deviation (SD), and a two-sided *p*-value of 0.05 or less was considered statistically significant. (*p < 0.05; **p < 0.01; ***p < 0.001; NS not significant).

### Reporting summary

Further information on research design is available in the Nature Research Reporting Summary linked to this article.

## Supplementary information

Supplementary information

Reporting Summary

Source Data

## Data Availability

The source data underlying Figs 2b–e, 3b, c, 5a, and 6c are provided as a Source Data file. All the other data that support the findings of this study are available from the corresponding author upon reasonable request.

## References

[1] Mamelle Gerard, Pampurik Jean, Luboinski Bernard, Lancar Remi, Lusinchi Antoine, Bosq Jacques (1994). Lymph node prognostic factors in head and neck squamous cell carcinomas. The American Journal of Surgery.

[2] Civantos FJ (2010). Sentinel lymph node biopsy accurately stages the regional lymph nodes for T1-T2 oral squamous cell carcinomas: results of a prospective multi-institutional trial. J. Clin. Oncol..

[3] National Comprehensive Cancer Network. NCCN Clinical Practice Guidelines in Oncology (NCCN Guidelines). Head and Neck Cancers. version 1 (2017).

[4] D’Cruz AK (2015). Elective versus therapeutic neck dissection in node-negative oral cancer. N. Engl. J. Med..

[5] Alkureishi LW (2010). Sentinel node biopsy in head and neck squamous cell cancer: 5-year follow-up of a European multicenter trial. Ann. Surg. Oncol..

[6] Gillies EM, Luna MA (1998). Histologic evaluation of neck dissection specimens. Otolaryngol. Clin. North. Am..

[7] Seethala RR (2009). Current state of neck dissection in the United States. Head. Neck Pathol..

[8] Rosenthal EL (2015). Safety and tumor specificity of cetuximab-IRDye800 for surgical navigation in head and neck cancer. Clin. Cancer Res..

[9] van Dam GM (2011). Intraoperative tumor-specific fluorescence imaging in ovarian cancer by folate receptor-α targeting: first in-human results. Nat. Med..

[10] Lamberts LE (2017). Tumor-specific uptake of fluorescent bevacizumab-IRDye800CW microdosing in patients with primary breast cancer: A phase I feasibility study. Clin. Cancer Res..

[11] Okusanya OT (2015). Intraoperative molecular imaging can identify lung adenocarcinomas during pulmonary resection. J. Thorac. Cardiovasc. Surg..

[12] Rosenthal EL (2017). Sensitivity and specificity of cetuximab-IRDye800CW to identify regional metastatic disease in head and neck cancer. Clin. Cancer Res..

[13] Gao RW (2018). Determination of tumor margins with surgical specimen mapping using near-infrared fluorescence. Cancer Res..

[14] Predina JD (2018). A phase I clinical trial of targeted intraoperative molecular imaging for pulmonary adenocarcinomas. Ann. Thorac. Surg..

[15] van Keulen S (2019). Rapid, non-invasive fluorescence margin assessment: Optical specimen mapping in oral squamous cell carcinoma. Oral. Oncol..

[16] Warram JM (2016). Fluorescence imaging to localize head and neck squamous cell carcinoma for enhanced pathological assessment. J. Pathol. Clin. Res..

[17] Carlson RO, Amirahmadi F, Hernandez JS (2012). A primer on the cost of quality for improvement of laboratory and pathology specimen processes. Am. J. Clin. Pathol..

[18] Wright FC, Law CH, Berry S, Smith AJ (2009). Clinically important aspects of lymph node assessment in colon cancer. J. Surg. Oncol..

[19] Cohen SM, Wexner SD, Schmitt SL, Nogueras JJ, Lucas FV (1994). Effect of xylene clearance of mesenteric fat on harvest of lymph nodes after colonic resection. Eur. J. Surg..

[20] Märkl B (2009). Methylene blue-assisted lymph node dissection in combination with ex vivo sentinel lymph node mapping in gastric cancer. Histopathology.

[21] Liberale G (2016). Ex vivo detection of tumoral lymph nodes of colorectal origin with fluorescence imaging after intraoperative intravenous injection of indocyanine green. J. Surg. Oncol..

[22] Pop CF (2018). Ex vivo indocyanine green fluorescence imaging for the detection of lymph node involvement in advanced-stage ovarian cancer. J. Surg. Oncol..

[23] Dahlberg AM (2014). The lymphatic system plays a major role in the intravenous and subcutaneous pharmacokinetics of trastuzumab in rats. Mol. Pharm..

[24] Yadav P (2018). Distribution of therapeutic proteins into thoracic lymph after intravenous administration is protein size-dependent and primarily occurs within the liver and mesentery. J. Control Release.

[25] Nishio N (2019). Optimal dosing strategy for fluorescence-guided surgery with panitumumab-IRDye800CW in head and neck cancer. Mol. Imaging Biol..

[26] DSouza AV, Lin H, Henderson ER, Samkoe KS, Pogue BW (2016). Review of fluorescence guided surgery systems: identification of key performance capabilities beyond indocyanine green imaging. J. Biomed. Opt..

[27] van den Berg NS (2012). Concomitant radio- and fluorescence-guided sentinel lymph node biopsy in squamous cell carcinoma of the oral cavity using ICG-(99m)Tc-nanocolloid. Eur. J. Nucl. Med. Mol. Imaging.

[28] Hekman MC (2018). Tumor-targeted dual-modality imaging to improve intraoperative visualization of clear cell renal cell carcinoma: A first in man study. Theranostics.

[29] Tichauer KM, Wang Y, Pogue BW, Liu JTC (2015). Quantitative in vivo cell-surface receptor imaging in oncology: kinetic modeling and paired-agent principles from nuclear medicine and optical imaging. Phys. Med. Biol..

[30] Tichauer KM (2014). Microscopic lymph node tumor burden quantified by macroscopic dual-tracer molecular imaging. Nat. Med..

[31] Li C (2019). Paired-agent fluorescence molecular imaging of sentinel lymph nodes using indocyanine green as a control agent for antibody-based targeted agents. Contrast Media Mol. Imaging.

[32] Pressman D, Day ED, Blau M (1957). The use of paired labeling in the determination of tumor-localizing antibodies. Cancer Res..

[33] Mallidi S (2009). Multiwavelength photoacoustic imaging and plasmon resonance coupling of gold nanoparticles for selective detection of cancer. Nano. Lett..

[34] Wang LV, Hu S (2012). Photoacoustic tomography: in vivo imaging from organelles to organs. Science.

[35] Valluru KS, Wilson KE, Willmann JK (2016). Photoacoustic imaging in oncology: translational preclinical and early clinical experience. Radiology.

[36] Upputuri PK, Pramanik M (2016). Recent advances toward preclinical and clinical translation of photoacoustic tomography: a review. J. Biomed. Opt..

[37] Stoffels I (2015). Metastatic status of sentinel lymph nodes in melanoma determined noninvasively with multispectral optoacoustic imaging. Sci. Transl. Med..

[38] Edge, S.B. et al. AJCC cancer staging handbook: from the AJCC cancer staging manual. 7th ed. New York, Springer-Verlag, 357–376 (2010).

[39] van Keulen S (2019). The Sentinel margin: intraoperative ex vivo specimen mapping using relative fluorescence intensity. Clin. Cancer Res..

