# Supplementary material for: Optical molecular imaging can differentiate metastatic from benign lymph nodes in head and neck cancer
Source: Nat Commun. 2019 Nov 6;10:5044. doi: 10.1038/s41467-019-13076-7 (PMC6834597; doi:10.1038/s41467-019-13076-7)
Supplement: Supplementary file 1 — Supplementary information [file 41467_2019_13076_MOESM1_ESM.pdf]

Supplementary information

Optical Molecular Imaging can Differentiate Metastatic from Benign Lymph Nodes in Head and Neck Cancer

Nishio et al.

Supplementary Table 1: Cumulative Statistics for the Total Number of Harvested LNs per Group

	Low dose (<0.5 mg kg^{-1})	Middle dose (≥0.5-<0.75 mg kg^{-1})	High dose (≥0.75 mg kg^{-1})
Number of LNs	287	236	489
Metastatic			
True positive	8	11	14
False negative	1	1	4
Benign			
False positive	6	7	45
True negative	272	217	426
Sensitivity	88.9%	91.7%	77.8%
Specificity	97.8%	96.9%	90.4%
Positive predictive value	57.1%	61.1%	23.7%
Negative predictive value	99.6%	99.5%	99.1%
Accuracy	97.6%	96.6%	90.0%

LN = lymph node.
