# Supplementary material for: Optical molecular imaging can differentiate metastatic from benign lymph nodes in head and neck cancer
Source: Nat Commun. 2019 Nov 6;10:5044. doi: 10.1038/s41467-019-13076-7 (PMC6834597; doi:10.1038/s41467-019-13076-7)
Supplement: Supplementary file 2 — Reporting Summary [file 41467_2019_13076_MOESM2_ESM.pdf]

nature research

Corresponding author(s): Eben L. Rosenthal

Last updated by author(s): 07/10/2019

# Reporting Summary

Nature Research wishes to improve the reproducibility of the work that we publish. This form provides structure for consistency and transparency in reporting. For further information on Nature Research policies, see Authors & Referees and the Editorial Policy Checklist .

## Statistics

For all statistical analyses, confirm that the following items are present in the figure legend, table legend, main text, or Methods section.

| n/a | Confirmed | |
|---|---|---|
| ☐ | ☒ | The exact sample size (*n*) for each experimental group/condition, given as a discrete number and unit of measurement |
| ☐ | ☒ | A statement on whether measurements were taken from distinct samples or whether the same sample was measured repeatedly |
| ☐ | ☒ | The statistical test(s) used AND whether they are one- or two-sided<br>*Only common tests should be described solely by name; describe more complex techniques in the Methods section.* |
| ☐ | ☒ | A description of all covariates tested |
| ☒ | ☐ | A description of any assumptions or corrections, such as tests of normality and adjustment for multiple comparisons |
| ☐ | ☒ | A full description of the statistical parameters including central tendency (e.g. means) or other basic estimates (e.g. regression coefficient) AND variation (e.g. standard deviation) or associated estimates of uncertainty (e.g. confidence intervals) |
| ☒ | ☐ | For null hypothesis testing, the test statistic (e.g. *F*, *t*, *r*) with confidence intervals, effect sizes, degrees of freedom and *P* value noted<br>*Give P values as exact values whenever suitable.* |
| ☒ | ☐ | For Bayesian analysis, information on the choice of priors and Markov chain Monte Carlo settings |
| ☒ | ☐ | For hierarchical and complex designs, identification of the appropriate level for tests and full reporting of outcomes |
| ☒ | ☐ | Estimates of effect sizes (e.g. Cohen's *d*, Pearson's *r*), indicating how they were calculated |

*Our web collection on statistics for biologists contains articles on many of the points above.*

## Software and code

Policy information about availability of computer code

| | |
|---|---|
| Data collection | N/A |
| Data analysis | Statistical analysis: Graphpad Prism (version 6.0c, GraphPad Software, La Jolla, CA, US); JMP (version 10, SAS, Cary NC, US); Microsoft Office Excell for generating data sheets; |

For manuscripts utilizing custom algorithms or software that are central to the research but not yet described in published literature, software must be made available to editors/reviewers. We strongly encourage code deposition in a community repository (e.g. GitHub). See the Nature Research guidelines for submitting code & software for further information.

## Data

Policy information about availability of data

All manuscripts must include a data availability statement. This statement should provide the following information, where applicable:

- Accession codes, unique identifiers, or web links for publicly available datasets
- A list of figures that have associated raw data
- A description of any restrictions on data availability

The source data underlying Figs 2b-e, 3b, c 5a, and 6c are provided as a Source Data file. All the other data that support the findings of this study are available from the corresponding author upon reasonable request.

# Field-specific reporting

Please select the one below that is the best fit for your research. If you are not sure, read the appropriate sections before making your selection.

# Life sciences study design

All studies must disclose on these points even when the disclosure is negative.

| | |
|---|---|
| Sample size | No sample-size calculation was performed. Sample size was chosen based on the number of patients included in our phase I study, at the time 24 patients. The sample size was considered sufficient for data evaluation. |
| Data exclusions | Of the 24 patients included in the study, patients not undergoing a loco-regional lymph node dissection were excluded from the study (n=2). |
| Replication | N/A. |
| Randomization | No randomization. |
| Blinding | No blinding. |

# Reporting for specific materials, systems and methods

We require information from authors about some types of materials, experimental systems and methods used in many studies. Here, indicate whether each material, system or method listed is relevant to your study. If you are not sure if a list item applies to your research, read the appropriate section before selecting a response.

## Materials & experimental systems

| n/a | Involved in the study | |
|---|---|---|
| ☐ | ☒ | Antibodies |
| ☒ | ☐ | Eukaryotic cell lines |
| ☒ | ☐ | Palaeontology |
| ☒ | ☐ | Animals and other organisms |
| ☐ | ☒ | Human research participants |
| ☐ | ☒ | Clinical data |

## Methods

| n/a | Involved in the study | |
|---|---|---|
| ☒ | ☐ | ChIP-seq |
| ☒ | ☐ | Flow cytometry |
| ☒ | ☐ | MRI-based neuroimaging |

# Antibodies

| | |
|---|---|
| Antibodies used | panitumumab-IRDye800CW (produced through NCI's NEXT program);<br>anti-EGFR antibody (clone EP38Y; lot no. 10128385; Thermo Fisher Scientific, Waltham, Massachusetts, USA);<br>anti-cytokeratin 5/6 antibody (clone D5/16B4; lot no. 20053896, Thermo Fisher Scientific, Waltham, Massachusetts, USA);<br>anti-CD68 antibody (clone KP1; lot. no. 20057124; Thermo Fisher Scientific, Waltham, Massachusetts, USA);<br>anti-CD31 antibody (ab124432; lot no. GR320647-4, Abcam, Cambridge, MA, USA). |
| Validation | Panitumumab-IRDye800CW was produced through NCI's NEXT program. Quality control included analysis of drug product in sterile vials for particulates and integrity of the sterilizing filter. Sterile via were transported to Stanford University under temperature controlled conditions. Vials are stored at Stanford, at the Stanford University Medical Center Investigational Pharmacy. A certificate of analysis was provided to us by NCI and stability tests are/were regularly performed to confirm product stability.<br><br>anti-EGFR antibody: https://static.thermoscientific.com/images/D12446~.pdf<br>anti-cytokeratin 5/6: https://www.thermofisher.com/antibody/product/Cytokeratin-5-6-Antibody-clone-D5-I6-B4-Monoclonal/MA5-12429<br>anti-CD68: https://www.thermofisher.com/antibody/product/CD68-Antibody-clone-KP1-Monoclonal/MA5-13324<br>anti-CD31: https://www.abcam.com/cd31-antibody-ab124432.html |

# Human research participants

Policy information about studies involving human research participants

| Field | Response |
|---|---|
| Population characteristics | Patients, M/F, >19 years of age, with biopsy-proven head and neck squamous cell carcinoma scheduled to undergo surgical resection of curative intent were eligible to participate in the study. A total of 24 patients were enrolled in the study of which 2 were excluded for further analysis as they did not undergo a neck dissection.<br><br>Characteristics of the remainder 22 patients are as follows: 16/22 patients were male. The average age was 60 years (range 32-85) and all patients had biopsy confirmed squamous cell carcinoma of the head and neck and were scheduled to undergo surgical resection of the primary tumor with subsequent loco-regional neck dissection. Twenty patients presented with an oral cavity tumor, one patient with squamous cell carcinoma of the hypopharynx and one patient with squamous cell carcinoma of the larynx. The clinical loco-regional lymph node status was as follows: 14 patients were clinically staged with an N0 neck, 5 patients were staged N1 and 3 patients N2. |
| Recruitment | Patients were recruited from the head and neck oncology practice at Stanford. All patients were evaluated by Dr. Rosenthal and a medical-oncologist (Dr. Colevas) to ensure that the patient met all eligibility criteria. Patients were initially approached by the protocol director, Dr. Rosenthal.<br><br>Written Informed Consent and HIPAA Authorization were obtained after the Informed Consent was reviewed and the study was fully explained to the patient including potential risks and discomforts – this was done by the clinical trial coordinators (Mr. Oberhelman and/or Mrs. Chirita). Full disclosure of the details and the investigational nature of the proposed protocols was provided by both the protocol director, Dr. Rosenthal and by the clinical trial coordinators.<br><br>The protocol adhered to regulations to provide protection for human subjects in clinical investigations described by the general requirements for informed consent. The DSMB of Stanford University provided oversight for the trial and trial safety.<br><br>Bias: To the best of our knowledge, self-selection bias did not occur. |
| Ethics oversight | Stanford University's IRB, the Administrative Panel on Human Subjects in Medical Research |

Note that full information on the approval of the study protocol must also be provided in the manuscript.

# Clinical data

Policy information about clinical studies

All manuscripts should comply with the ICMJEguidelines for publication of clinical research and a completedCONSORT checklist must be included with all submissions.

| Field | Response |
|---|---|
| Clinical trial registration | NCT02415881 |
| Study protocol | The study protocol (Stanford IRB-35064) is available from the corresponding author upon reasonable request. |
| Data collection | Enrolled patients underwent surgery between 12/2015-3/2018. On the day of surgery, fluorescence imaging data was gathered in the operation theatre. Subsequently, fluorescence imaging data was gathered during the pathological processing steps; this data was collected in the Rosenthal Laboratory at Stanford University. Data analysis, including fluroscence imaging data analysis, immunohistochemistry and statistical analysis was performed between 3/2018 and 1/2019 in the Rosenthal Laboratory at Stanford University. |
| Outcomes | Primary outcome: Number of panitumumab-IRDye800 positive lymph nodes as determined by ex vivo fluorescence imaging using a closed-field device that are also tumor-positive at (histo-) pathology.<br>Assessed as follows: At pathology all lymph nodes were harvested from the surgically removed tissue specimens (i.e. loco-regional lymph node dissection specimens). Hereafter, fluorescence imaging of these nodes was performed on the closed-field imaging device and mean fluorescence intensities of the individual lymph nodes was determined (in arbitrary units). Subsequently the nodes were processed and evaluated by a board-certified pathologist as such to determine whether or not these nodes were tumor-positive. Fluorescence imaging results were compared to pathology findings.<br><br>Secondary outcome: Sensitivity and specificity of panitumumab-IRDye800 for metastatic lymph node identification.<br>Assessed as follows: Similar as above, at pathology all lymph nodes were harvested from the surgically removed tissue specimens (i.e. loco-regional lymph node dissection specimens). Hereafter, fluorescence imaging of these nodes was performed on the closed-field imaging device and mean fluorescence intensities of the individual lymph nodes was determined (in arbitrary units). Subsequently the nodes were processed and evaluated by a boar-certified pathologist as such to determine whether or not these nodes were tumor-positive. Subsequently, mean fluorescence intensity readings and pathological outcomes were loaded into Graphpad as such to determine the sensitivity, specificity, negative and positive predictive value of the approach.<br><br>Tertiary outcome: Mean fluorescence intensity and signal-to-background ratio threshold at which the highest sensitivity and specificity of panitumumab-IRDye800 for metastatic lymph node identification is reached.<br>Assessed as follows: Similar as above, at pathology all lymph nodes were harvested from the surgically removed tissue specimens (i.e. loco-regional lymph node dissection specimens). Hereafter, fluorescence imaging of these nodes was performed on the closed-field imaging device and mean fluorescence intensities of the individual lymph nodes was determined (in arbitrary units). Subsequently the nodes were processed and evaluated by a boar-certified pathologist as such to determine whether or not these nodes were tumor-positive. Subsequently, mean fluorescence intensity readings and pathological outcomes were loaded |

into Graphpad as such to determine the value where sensitivity, specificity, negative and positive predictive value of the approach was highest.

To the determine the signal-to-background ratio threshold, as background reading the mean fluorescence intensity of surrounding adipose tissue was chosen (lymph nodes are generally embedded in adipose tissue). Signal-to-background ratio's were calculated by dividing the mean fluorescence intensity signal of the lymph node by that of the adipose tissue. Subsequently, obtained signal-to-background ratio's and pathological outcomes of the individual lymph nodes were loaded into Graphpad as such to determine the value where sensitivity, specificity, negative and positive predictive value of the approach was highest.

Quaternary: The minimum number of fluorescence-positive lymph nodes that have to be assessed in order to achieve accurate nodal staging (i.e. N-stage in TNM-staging (AJCC, 7th edition).
Assessed as follows: At pathology all lymph nodes were harvested from the surgically removed tissue specimens (i.e. loco-regional lymph node dissection specimens). Hereafter, fluorescence imaging of these nodes was performed on the closed-field imaging device and mean fluorescence intensities of the individual lymph nodes was determined (in arbitrary units). Subsequently the nodes were processed and evaluated by a board-certified pathologist as such to determine whether or not these nodes were tumor-positive. Fluorescence imaging results were compared to pathology findings. Hereafter, all lymph nodes were ranked based on their mean fluorescence intensity from high mean fluorescence intensity to low mean fluorescence intensity. From each patient it was then noted how many fluorescence-positive lymph nodes per neck should be evaluated in order to accurately stage the neck of the patient. This information was then loaded into Graphpad and the overall number of nodes to be assessed per neck to accurately stage the patient was determined.
